# Comparison of the effects of abdominal massage and osteopathic manipulative treatment home program on constipation in children with cerebral palsy

**DOI:** 10.1002/jgh3.13102

**Published:** 2024-06-19

**Authors:** Aisel T Chatip, Gönül Acar, Ayfer A Akçay

**Affiliations:** ^1^ Yıldız Special Education and Rehabilitation Center for Children Istanbul Turkey; ^2^ Department of Physiotherapy and Rehabilitation Marmara University Faculty of Health Sciences Istanbul Turkey; ^3^ Koç University Hospital Istanbul Turkey

**Keywords:** abdominal massage, cerebral palsy, constipation, osteopathic manipulative treatment, physiotherapy

## Abstract

**Background and Aim:**

The aim of this study is to compare the effects of osteopathic manipulative therapy home program (OMT‐H) *versus* abdominal massage home program (AMHP) in treating constipation in children with cerebral palsy (CP).

**Methods:**

Twenty‐nine children with CP with a mean age of 12.2 ± 3.76 years, who were constipated and were not on medication, were divided into three randomized groups: (i) control group (*n* = 10), (ii) AMHP (*n* = 10), and (iii) OMT‐H (*n* = 9). In AMHP and OMT‐H groups, treatment was applied as 20‐min sessions every other day for 10 sessions for 3 weeks. Modified Constipation Assessment Scale (MCAS), Rome III criteria, and the Bristol Stool Form Scale (BSFS) were used for evaluation before treatment and once a week during treatment.

**Results:**

While there was no change in constipation symptoms in the control group, there was an improvement in constipation symptoms after treatment in the AMHP and OMT‐H groups (AMHP, *P* = 0.003; OMT‐H, *P* = 0.000014). While the treatment showed to be effective from the first week in the OMT‐H group, the change in BSFS (*P* = 0.026) and MCAS sub‐parameters was found to be superior.

**Conclusion:**

AMHP and OMT‐H are effective and beneficial in treating constipation. In children with CP, OMT‐H was found to be quicker and more successful compared with AMHP. The OMT‐H can be effectively used in clinical practice in relieving constipation in CP.

## Graphical abstract

In our study to investigate the best approach to the problem of constipation, which complicates daily life in children with cerebral palsy, and to evaluate the applicability of these treatments by mothers/caregivers at home, we examined the symptoms seen in children; Complaints such as stool consistency, frequency of defecation, abdominal bloating, and inability to pass stool resolved in a short time. Osteopathic treatment is considered a useful and effective approach in the treatment of constipation in cerebral palsy.

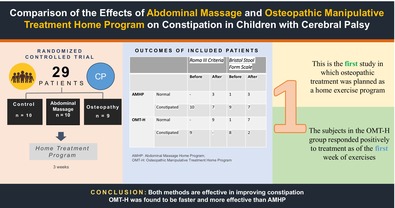

## Introduction

Cerebral palsy (CP) is defined as a group of persistent but non‐progressive disorders of the development of movement and posture, resulting from damage that occurred in the developing fetal or infant brain. In CP, the motor disorders of the disease often are accompanied by disturbances in sensation, perception, cognition, communication, behavior, epilepsy, and secondary musculoskeletal problems.^1^ Children with chronic disabilities due to neurodevelopmental disorders, depending on the severity of the disease, frequently experience gastrointestinal system disorders.^2^ The prevalence of constipation in children with CP ranges from 26% to 74% in studies conducted.^3^, ^4^, ^5^ Causes such as increased muscle tone, decreased defecation, immobilization, mental retardation, poor diet, and malnutrition reduce the frequency of defecation, resulting in constipation.^6^ Decreased colonic motility and changes in neural modulation are also thought to cause constipation.^7^ While defecation frequency is three to four times a week in healthy children, it is once a week or once every 10 days in children with CP.^7^, ^8^ Children with CP have feeding problems, which cause lower energy and nutrient intakes and malnutrition.^5^ Methods used in the treatment of constipation in children with CP include fluid supplementation,^7^ high‐fiber diet,^8^, ^9^ biofeedback, oral laxatives and rectal stimulants,^2^, ^3^, ^10^ prebiotics,^11^ hypnotherapy,^12^ chiropractic methods,^13^, ^14^ reflexology,^13^, ^15^ connective tissue massage, kinesiology taping,^16^ sacral neuromodulation,^17^ abdominal massage,^18^ and osteopathy.^19^

Abdominal massage is the most commonly used noninvasive method in constipation, and this method strengthens the gastrocolic reflex by applying pressure on the abdominal wall with movements, triggers receptors, and provides stimulation and relaxation.^11^, ^12^, ^20^, ^21^, ^22^ There are different abdominal massage techniques used in pediatric diseases^12^; however, only a few studies have shown the effectiveness of abdominal massage in children with neurological damage.^16^, ^22^, ^23^

Osteopathic manipulative therapy (OMT) is a diagnostic and therapeutic method that holistically considers the movement of tissues and organs and their interaction with one another, argues that such movement is important for health and healing processes, and that mobilizes and applies manipulations to the musculoskeletal and visceral systems for treatment.^24^, ^25^, ^26^ In OMT, the free movement between the tissues is considered important for the continuity of health and healing processes, and the practitioner mobilizes and applies manipulations to the musculoskeletal and visceral systems.^24^, ^26^, ^27^, ^28^ The purpose of using OMT in constipation is to regulate the movement between the abdominal organs as well as the movement within the organs themselves.^9^ A limited number of studies have reported OMT as an effective treatment method in children with neurological damage.^9^, ^19^, ^25^, ^29^, ^30^, ^31^

In children with CP, therapies applied by laypersons other than physiotherapists, including parents, caregivers, and assistants, are essential, but therapies not performed by experienced specialists are considered less reliable from a scientific point of view. However, it is not possible to apply all therapies by a physiotherapist in clinical practice. Also, home exercises have become an integral part of the treatment in our country. Under today's pandemic conditions, training the mother/caregiver for a home program planned by the physiotherapist and making telephone calls/online follow‐up in patients with CP have become a part of our clinical practice.^32^, ^33^

Although constipation in children with CP is a nonmotor problem that negatively affects the quality of life of both the child and the family,^34^ the studies addressing the topic are not enough. Abdominal massage home program (AMHP) has been used since years, while OMT is a relatively new method. Our study aims to compare the effect of AMHP and osteopathic manipulative therapy home program (OMT‐H) applied in children with CP who had constipation in relieving constipation and to investigate the feasibility and effectiveness of OMT as a home program.

## Materials and methods

The ethical approval of the study was obtained from Marmara University Clinical Trials Ethics Committee with protocol code 09.2019.496 dated 3 April 2019. Of 30 patients admitted between May 2019 and December 2019, 29 were included in the study. The study was conducted as a prospective and randomized controlled study. Written informed consents were received from the parents of the subjects.

### Patient selection

The study included children with constipation who had previously received treatment but stopped because it did not benefit them or those who refused medication; thus, the results of the treatment we provided would not be affected by external factors such as diet and medication.

Thirty children with constipation who had CP but no other comorbidities and those who were not on medication or a special diet were divided into three groups: (i) control group (CG) (*n* = 10), (ii) AMHP (*n* = 10), and (iii) OMT‐H (*n* = 9). However, one subject who did not keep up with the program for 1 week or longer was excluded from the study.

### Compliance with ethical standards

The authors report no conflicts of interest, financial, or otherwise. The ethical approval of the study was obtained from Marmara University Clinical Trials Ethics Committee and written informed consents were received from the parents of the subjects.

### Treatment

OMT‐H included sacrum oscillation, reflex point therapy, diaphragm technique (lower rib mobilization), and rib‐rising technique, while AMHP included traditional bowel massage. No intervention was made for constipation in the control group and children in all groups continued their routine physical therapy sessions. For subjects in OMT‐H and AMHP groups, the programs were taught to the mother/caregiver as a home program, and they were allowed to take video recordings of the sessions with their mobile phones. Parents were monitored throughout one session, and accordingly, a checklist was completed to ensure that they apply the treatment correctly. In AMHP and OMT‐H groups, treatment was applied as 20‐min sessions every other day for a total of 10 sessions over 3 weeks. The subjects were evaluated once a week during the 3 weeks, and it was queried whether there was a problem with the OMT application or not.

### Evaluation parameters

Sociodemographic characteristics of the children with CP included in the study were recorded. Rome III criteria were used to evaluate diagnosis and treatment results. The Bristol Stool Form Scale was used to determine stool consistency. The Modified Constipation Assessment Scale was used to assess constipation status.^35^ Pedia and physiotherapist asked all evaluation scales, and the parameters were explained to the parents one by one and recorded. Changes were evaluated and recorded at the beginning of the study, and changes were also recorded at the end of the first, second, and third weeks. MCAS was scored as “0” for no problem, “1” for some problem, and “2” for severe problem. Then, total MCAS values were recorded weekly in all patients, and the change after treatment was examined as a percentage, and the change of each sub‐parameter after treatment was recorded statistically. Changes in Rome III criteria and BSFS were evaluated and recorded at the beginning and end of the study.

On the evaluation days, parents repeated the treatment they applied at home, accompanied by a physiotherapist, to ensure that the taught practices were carried out correctly. At the same time, they were examined by phone once a week and asked whether they had any problems with the treatment they received.

### Statistical analysis

The study data were analyzed using the SPSS 17.0, and the distribution of variables was described using numbers, percentages, mean, and SD. The Kruskal–Wallis test was used for the comparison of the scale scores between the groups. The Wilcoxon test was used to compare the baseline and end‐of‐study scores of nonparametric tests, whereas the Friedman test was used to compare the weekly changes. A significance level of *P* < 0.05 was used for the study data.

## Results

The average age of the cases was 12.2 ± 3.76 years (the youngest was 7, the oldest was 17). It was determined that the age, height, weight, and BMI of the cases were normally distributed, but CP types and GMCFS levels were not (*P* > 0.05). The results are shown in Table 1.

**Table 1:** Sociodemographic characteristics of patients

		CG mean ± SD	AMHP mean ± SD	OMT‐H mean ± SD	*P*
Age (mean ± SD)		11.89 ± 3.48	11.4 ± 4.24	14.2 ± 5.39	0.418
Gender	Boy	5	4	5	
	Girl	5	6	4	
Height, cm (mean ± SD)		145 ± 11	140 ± 21	149 ± 16	0.262
Weight, kg (mean ± SD)		42.44 ± 12.79	40.8 ± 15.77	46.1 ± 17.7	0.628
BMI, kg/m^2^ (mean ± SD)		19.68 ± 3.64	19.65 ± 4.3	19.89 ± 5.3	0.71
CP type, *n* (%)	Hemiparesis	4 (40)	3 (30)	3 (30)	<0.05
	Diparesis	3 (30)	3 (30)	4 (40)	
	Tetraparesis	1 (20)	2 (40)	2 (40)	
	Ataxic	0 (0)	1 (100)	0 (0)	
	Athetoid	2 (66.7)	1 (33.3)	0 (0)	
GMFCS	Level I	3	3	—	<0.05
	Level II	3	3	5	
	Level III	1	1	3	
	Level IV	1	—	—	
	Level V	2	3	1	

AMHP, Group Receiving the Abdominal Massage Home Program; BMI, body mass index; CG, control group; CP, cerebral palsy; GMFCS, Gross Motor Function Classification System; OMT‐H, Group Receiving the Osteopathic Manipulative Treatment Home Program.

Considering the MKDS parameters and distribution of the cases, 65% of the cases (*n* = 19) had mild or severe abdominal pain and bloating, 79% (*n* = 23) had rectal fullness or pressure, 37% (*n* = 11) had low‐volume stools, 58% (*n* = 17) had high‐volume stools, and 86% (*n* = 25) had the problem of inability to pass stool. When the difference between the mean values of MCAS was evaluated, there was a significant change in the OMT‐H group in the first week, while a significant change was observed in the AMHP group at the end of Week 3 (*P* = 0.024, *P* = 0.027, respectively) (Table 2).

**Table 2:** Weekly changes in the Modified Constipation Assessment Scale by groups

	CG			AMHP			OMT‐H		
	Mean ± SD		*P*	Mean ± SD		*P*	Mean ± SD		*P*
Before treatment	5	2.7		7	3.23		9.11	3.33	
Week 1	5	2.58	1.0	6.5	2.63	0.357	5.66	3.9	**0.007**
Week 2	4.8	2.61	0.15	5.8	2.85	0.059	3.33	3.87	**0.016**
Week 3	4.7	2.71	0.56	4.6	3.13	**0.024**	1.55	1.94	**0.027**

While a positive response to the treatment was obtained in the osteopathic treatment group from first week; In abdominal massage group, this response was obtained only in the 3th week.

AMHP, Group Receiving the Abdominal Massage Home Program; CG, control group; OMT‐H, Group Receiving the Osteopathic Manipulative Treatment Home Program.

When we examined MCAS parameters individually, there was an improvement in a higher number of parameters in the OMT‐H group than in the AMHP group (7 against 2) (Table 3).

**Table 3:** Intergroup comparison of the Modified Constipation Assessment Scale sub‐parameters

	CG (mean) *n* = 10			AMHP (mean) *n* = 10			OMT‐H (mean) *n* = 9		
	BT	AT	*P*	BT	AT	*P*	BT	AT	*P*
1‐Abdominal distension or bloating	0.6	0.6	1	1	0.4	**0.034**	0.89	0	**0.023**
2‐Change in amount of gas passed rectally	0.2	0.3	0.317	0.6	0.2	0.102	0.66	0	**0.014**
3‐Less frequent bowel movements	0.7	0.7	1	0.8	0.5	0.257	1	0.11	**0.005**
4‐More frequent bowel movements	0.7	0.6	0.317	0.9	0.6	0.18	1.11	0.11	**0.007**
5‐Oozing liquid stool	0.1	0.1	1	0	0	1	0	0	1
6‐Rectal fullness or pressure	0.7	0.5	0.157	1.2	0.9	0.083	1.55	0.77	**0.02**
7‐Smaller stool size	0.4	0.4	1	0.2	0.2	1	0.55	0	0.157
8‐Bigger stool size	0.8	0.8	1	1.3	0.9	**0.046**	1.77	0.33	**0.006**
9‐Inability to pass the stool	0.8	0.7	0.317	1	0.9	0.317	1.55	0.22	**0.006**

The osteopathic group is superior in improving the factors of abdominal or bloating, change in the amount of rectal burping, decreased bowel motility, increased bowel motility, rectal fullness or pressure sensation, high volume stool and inability to pass stool.

AMHP, Group in the Abdominal Massage Home Program; AT, after treatment; BT, before treatment; CG, control group; OMT‐H, Group Receiving the Osteopathic Manipulative Treatment Home Program.

While all children were diagnosed with constipation according to the Rome III criteria before treatment, constipation was relieved in three children in the AMHP group (*P* = 0.25) and all children in the OMT‐H group (*P* = 0.004) after treatment. There was no change in the control group. When the subjects were assessed with the Bristol Stool Form Scale, stool consistency returned to normal in two children in the AMHP group (*P* = 0.05) and six children in the OMT‐H group at the end of the treatment (*P* = 0.016) (Table 4).

**Table 4:** Intergroup comparison of Roma III criteria and Bristol Stool Form Scale results

	Roma III criteria			Bristol Stool Form Scale		
	BT	AT	*P*	BT	AT	*P*
CG						
Normal	—	—	1	2	2	1
Constipated	10	10		8	8	
AMHP						
Normal	—	3	0.25	1	3	0.5
Constipated	10	7		9	7	
OMT‐H						
Normal	—	9	**0.004**	1	7	**0.016**
Constipated	9	—		8	2	

Before treatment, all children were diagnosed with constipation according to ROME III criteria; After treatment, the diagnosis of constipation was eliminated in 3 children in the abdominal massage group and in all children in osteopathy group.

At the Bristol stool scale, the stool consistency of 2 children in the abdominal massage group and 6 children in osteopathy group returned to normal.

AMHP, Group in the Abdominal Massage Home Program; AT, after treatment; BT, before treatment; CG, control group; OMT‐H, Group Receiving the Osteopathic Manipulative Treatment Home Program.

## Discussion

This is the first study in which osteopathic treatment was planned as a home exercise program, and it has demonstrated that abdominal massage and osteopathic home exercise programs were effective in improving constipation in CP. While the subjects in the OMT‐H group responded positively to treatment as of the first week of exercises, the response to treatment was obtained only in the third week in the AMHP group. In the OMT‐H group, stool consistency was improved in more children, and the diagnosis of constipation was cleared up after treatment according to the Rome III criteria. When we examined MCAS parameters individually, we observed improvement in a greater number of parameters in the OMT‐H group.

In the literature and clinical practice, constipation is mostly treated by combining the treatments that are thought to be beneficial. These combinations often include changes in the individual's diet and lifestyle.^18^, ^23^, ^36^ Studies have shown that increased intake of fibrous food and water consumption is effective in the treatment of constipation.^3^, ^7^, ^8^ Because our study aimed to compare therapy techniques, the subjects did not receive any additional nutrients and medication. Abdominal massage is the most commonly used method for relieving constipation. Many studies have emphasized that abdominal massage is an effective, easy, and inexpensive technique in the treatment of constipation.^11^, ^18^, ^37^ In their systematic review, Ernst *et al*. reported that abdominal massage is beneficial for chronic constipation. Moreover, Lamas *et al*. emphasized that abdominal massage can be used as a complementary therapy in cases where the use of laxatives is insufficient for treatment, and that the application of abdominal massage as a home exercise is an effective and low‐cost solution.^38^ In the pediatric disabled patient group, abdominal massage and diet modification improved constipation.^39^, ^40^ In a study where nutrition programs were implemented in addition to abdominal massage in the treatment of 50 children with quadriplegic CP, 90% of the subjects showed improvement in the aspects of quality of life, such as sleep, appetite, and irritability as well as rectal bleeding, anal fissure, crying, and pain during defecation. In that study, only 10% required laxatives.^23^ In our study, abdominal massage was performed as a 3‐week home program, and at the end of the study, 3 out of 10 children with CP in the AMHP group were no longer diagnosed with constipation.

Studies investigating the efficacy of OMT on constipation in different diseases are mainly adult studies,^30^, ^36^, ^37^, ^40^, ^41^, ^42^, ^43^, ^44^, ^45^ and studies involving pediatric age group are very limited.^46^, ^47^, ^48^, ^49^ Tarsuslu *et al*. emphasized that osteopathic methods are useful for the treatment of constipation in children with CP,^19^ and Zollars *et al*. stated that osteopathic methods improve the quality of life and can provide clinicians and families with an alternative approach to medications.^50^ The osteopathic techniques we used in this study were different from the other studies, and although the techniques were applied as a home program, positive results could be obtained as of the first week in reducing constipation in CP. In our study, mothers/caregivers emphasized that they could easily learn the techniques and that records on mobile phones were a good learning resource for them, and they were satisfied with the home program.

A study investigating the effects of different exercises other than massage techniques in the treatment of constipation in children with CP showed that there is a strong correlation between the level of spasticity and constipation in children with CP, and that stretching exercises applied daily to the lower and upper extremities with five repetitions and for 30 s during 6 weeks for the treatment of spasticity improved the symptoms of constipation.^46^ In a study, Tarsuslu *et al*. compared osteopathic treatment and osteopathic treatment plus medication on 13 children with CP with constipation; the subjects were evaluated at the third month and sixth month via the GMFCS, Modified Ashworth Scale, Functional Independence Measure, Constipation Assessment Scale, and Visual Analogue Scale. In both groups, there was an increase in the frequency of defecation (4–7 times a week) and an increase in family satisfaction, but no superiority was observed between the groups. Tarsuslu *et al*. emphasized that OMT is an effective method in the treatment of constipation in children with CP.^19^ The OMT techniques employed by Tarsuslu *et al*. are different from the techniques we used in our study. We consider our study to be superior to others in the way that, with no nutritional supplementation, there was a decrease in the complaints related to the MCAS parameters from the first week, stool consistency improved at the end of the third week, and the diagnosis of constipation was eliminated according to the Rome III criteria for all patients in the OMT‐H group, and that we trained the mothers/caregivers to apply the osteopathic approaches used in our study at home. In our study, the improvement results of constipation, especially in the OMT‐H group, were reported as verbal satisfaction of parents and children. However, not applying quality of life scale for the families and children is a limitation of the study.

BSFS, CAS, bowel diary, Rome III criteria, and MCAS^13^ are frequently used to evaluate constipation in CP. The Rome III criteria are the most commonly used scale for diagnosis.^3^, ^24^, ^41^, ^45^, ^47^, ^48^ BSFS is the seven‐item scale used to determine stool consistency in the studies.^39^, ^41^, ^47^, ^51^ CAS questions the presence and severity of constipation.^19^, ^46^ The bowel diary is widely used to question and record the frequency of defecation.^19^, ^24^, ^45^, ^47^, ^51^, ^52^ MCAS, on the other hand, questions complaints that may accompany constipation.^13^ In our study, the diagnosis of constipation was standardized for all children using the Rome III criteria. According to our literature review, there is no other study in the literature evaluating MCAS parameters individually. Evaluating the sub‐parameters enabled the assessment of the efficacy of the treatment, the implementation of approaches to the symptoms, and the adjustment of the treatment according to the needs of the children, and helped us see the differences between the two methods in more detail. In our study, there was a weekly modification in MCAS parameters as of the first week in the OMT‐H group, whereas the subjects in the AMHP group started experiencing changes in those parameters only at the end of the third week. While seven of nine complaints accompanying constipation in the OMT‐H group improved, only two of nine complaints improved in the AMHP group. Using MCAS made it possible to understand that OMT‐H can cure constipation faster.

It is emphasized in the literature that the complaints of constipation are more common in patients with CP who are more severe cases according to GMFCS and who have no ambulation ability. In the study by Tarsuslu *et al*., 83% of the patients were Level IV and Level V, according to GMFCS. It was reported in that study that the prevalence of constipation in 152 patients with CP, 83% of the patients had tetraparesis and 62% were suffering from constipation.^53^ Unlike those studies, in our study, 24% of the patients were at levels IV and V; most of them were at levels I, II, and III. In our cases consisting of this category, it has been demonstrated that constipation can also occur in children with CP who are more independent in movement. This indicates that the cause of constipation is not only due to immobilization but rather is a multifactorial condition. Because we included all CP children with constipation in our study and 68% (*n* = 20) of these cases had hemiparesis and diparesis, we cannot make a comment about the frequency of constipation in CP subtypes. In our study, the presence of constipation in mild types of CP other than the cases with tetraparesis suggested that, in contrary to the literature, all cases should be screened for constipation and families should be questioned in this regard. Because the number of these subtypes of CP was very low in our study, it is insufficient to investigate which approaches are more effective in improving constipation within this group.

## Conclusion

Treating constipation in CP as soon as possible and reducing complaints are important for improving the quality of life of the child and the family. This study was conducted to investigate the effects of abdominal massage and osteopathic techniques on constipation in CP.

Our study emphasizes that it offers a noninvasive and inexpensive method that can be applied at home to children with CP who have constipation, and that it will positively support and affect rehabilitation and also provide recovery in a short time. As a result of our examinations, it was found that both abdominal massage and osteopathic home exercise program are effective methods in improving constipation, and that the number of defecations increased with both methods, but the osteopathic home exercise group was superior in improving stool consistency. However, while a positive response to the treatment was obtained in the osteopathic home exercise group from the first week, in the abdominal massage group, this response was obtained only in the third week. In this regard, both methods have seen their effects in a short time, showing that OMT provides healing in a shorter time, starting from the first week.

MKDS parameters that we examined statistically one by one, osteopathic home exercise program was found to be more effective in reducing abdominal bloating or bloating, change in the amount of rectal gas passing, decrease in intestinal motility, increase in intestinal motility, feeling of rectal fullness or pressure, high‐volume stool, and inability to pass stool. All cases in the osteopathic home exercise group were excluded from the diagnosis of constipation according to the Rome III criteria after treatment. In light of the data obtained, osteopathic (rib‐rising, sacrum oscillation, diaphragm mobilization, and reflex point therapy) techniques have proven to be useful techniques in constipation; therefore, we believe that osteopathic approaches should be added to the treatment program in constipation in CP.

In our study, 24% of the patients were at levels IV and V, and the majority were at levels I, II, and III. It has been shown that constipation can also be seen in our cases consisting of this category, that is, children with CP who move more independently, and that the cause of constipation is not only due to immobilization, but that this is a multifactorial condition. Therefore, we recommend that all cases with CP should be screened for constipation and families should be questioned in this regard.

In short, our study was the first study in which osteopathic treatment was given as a home exercise to reduce constipation in CP. The recording feature of mobile phones has contributed to correct treatment and weekly supervision and telephone calls to families provided cost‐effective remote supervision. On the other hand, the use of a home exercise program checklist positively affected the application of the treatment at home.

We believe that the comparison of two approaches to constipation in a randomized and controlled manner will shed light and guide the literature clinically in terms of showing the benefit from the first week in the osteopathic home exercise group.

In the future, studies should be conducted with more cases in all types of CP, and the effectiveness of studies carried out together with nutritional counseling should be determined. We think that further studies with larger samples are needed.

## Ethical approval

All procedures performed in studies involving human participants were in accordance with the ethical standards of the institutional research committee and with the 1964 Helsinki declaration and its later amendments or comparable ethical standards.

## Informed consent

Informed consent was obtained from the parents of participants who participated in this study.
